# Cleft closure and other predictors of contemporary outcomes after atrioventricular canal repair in patients with parachute left atrioventricular valve

**DOI:** 10.1093/icvts/ivae048

**Published:** 2024-03-27

**Authors:** Patrick B McGeoghegan, Minmin Lu, Lynn A Sleeper, Sitaram M Emani, Christopher W Baird, Eric N Feins, Laura A Gellis, Kevin G Friedman

**Affiliations:** Department of Cardiology, Children’s Hospital Boston, Boston, MA, USA; Department of Cardiology, Children’s Hospital Boston, Boston, MA, USA; Department of Cardiology, Children’s Hospital Boston, Boston, MA, USA; Department of Cardiothoracic Surgery, Children’s Hospital Boston, Boston, MA, USA; Department of Cardiothoracic Surgery, Children’s Hospital Boston, Boston, MA, USA; Department of Cardiothoracic Surgery, Children’s Hospital Boston, Boston, MA, USA; Department of Cardiology, Children’s Hospital Boston, Boston, MA, USA; Department of Cardiology, Children’s Hospital Boston, Boston, MA, USA

**Keywords:** Parachute, Left atrioventricular valve, Atrioventricular septal defect, Cleft

## Abstract

**OBJECTIVES:**

Parachute left atrioventricular valve (LAVV) complicates atrioventricular septal defect (AVSD) repair. We evaluate outcomes of AVSD patients with parachute LAVV and identify risk factors for adverse outcomes.

**METHODS:**

We evaluated all patients undergoing repair of AVSD with parachute LAVV from 2012 to 2021. The primary outcome was a composite of time-to-death, LAVV reintervention and development of greater than or equal to moderate LAVV dysfunction (greater than or equal to moderate LAVV stenosis and/or LAVV regurgitation). Event-free survival for the composite outcome was estimated using Kaplan–Meier methodology and competing risks analysis. Cox proportional hazards regression was used to identify predictors of the primary outcome.

**RESULTS:**

A total of 36 patients were included with a median age at repair of 4 months (interquartile range 2.3–5.5 months). Over a median follow-up of 2.6 years (interquartile range 1.0–5.6 years), 6 (17%) patients underwent LAVV reintervention. All 6 patients who underwent LAVV reintervention had right-dominant AVSD. Sixteen patients (44%) met the composite outcome, and all did so within 2 years of initial repair. Transitional AVSD (versus complete), prior single-ventricle palliation, leaving the cleft completely open and greater than or equal to moderate preoperative LAVV regurgitation were associated with a higher risk of LAVV reintervention in univariate analysis. In multivariate analysis, leaving the cleft completely open was associated with the composite outcome.

**CONCLUSIONS:**

Repair of AVSD with parachute LAVV remains a challenge with a significant burden of LAVV reintervention and dysfunction in medium-term follow-up. Unbalanced, right-dominant AVSDs are at higher risk for LAVV reintervention. Leaving the cleft completely open might independently predict poor overall outcomes and should be avoided when possible.

**Clinical trial registration number:**

IRB-P00041642

**Figure:**
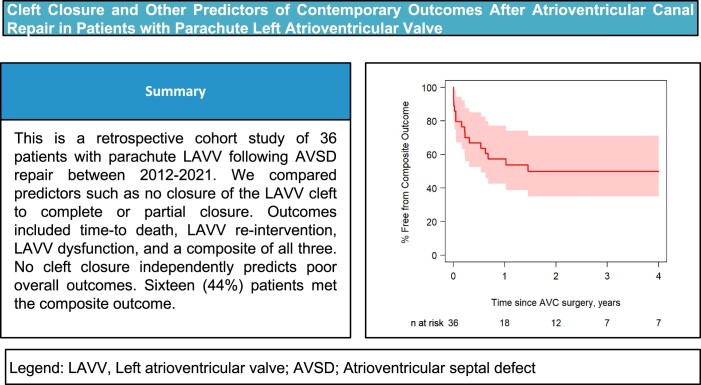

## INTRODUCTION

Repair of atrioventricular septal defects (AVSDs) is now routinely performed in infancy with good surgical outcomes and a low risk of reoperation [1–3]. In several large cohort studies, freedom from reoperation following AVSD repair is ∼85% at 10–20 years [1, 2, 4]. Left atrioventricular valve regurgitation (LAVVR) is the most common indication for repeat surgery [4–6], and anomalies of the left atrioventricular valve (LAVV) can complicate repair [6, 7]. Patients with AVSD and LAVV leaflets supported by a single papillary muscle (parachute LAVV) or 2 closely spaced papillary muscles and maldistribution of chordae (*forme fruste* parachute) are at higher risk for postoperative left atrioventricular valve stenosis (LAVVS), LAVVR and future need for LAVV repair or replacement [8, 9]. In the setting of a parachute LAVV, closure of the cleft can result in stenosis, while leaving the cleft open can predispose to LAVVR [8–10]. A single-centre study evaluating patients with complete AVSD and parachute LAVV undergoing repair between 2001 and 2012 found a high burden of reoperation (28% at 4 years) [9]. These patients may require a series of LAVV interventions to address residual LAVV disease [11, 12]. Faced with a parachute-type LAVV in AVSD, some institutions choose to abandon biventricular repair due to concerns about valve function and opt for single-ventricle palliation [9, 13]. Given the challenges in single-ventricle palliation [14–16], it is important to provide contemporary surgical outcomes of biventricular repair for patients with AVSD and parachute LAVV. Further, anatomic, echocardiographic and surgical predictors of adverse cardiac outcomes are scarce and may benefit clinical management of this patient population. Hence, the aims of the current study were to: (i) to describe contemporary medium-term postoperative outcomes following AVSD repair in patients with parachute LAVV, and (ii) identify factors associated with these adverse outcomes after repair.

## METHODS

### Ethics statement

This study was approved by the Boston Children’s Hospital Institutional Review Board (IRB-P00041642) on 3 March 2022. Patient written informed consent was waived by our Institutional Review Board, and this study complies with the ethical principles of the Declaration of Helsinki.

### Patient population

We included patients with AVSD and either parachute or *forme fruste* parachute LAVV who underwent full biventricular repair at Boston Children’s Hospital between 1 January 2012 and 1 January 2021. This included patients who underwent full repair of balanced or unbalanced AVSD and complete, transitional or partial defects. In this cohort, however, there were no patients with partial AVSD. Exclusion criteria included AVSD septation as part of staged left ventricular recruitment, double-orifice LAVV or clinically significant pulmonary vein stenosis. The primary outcome was a composite of death, LAVV reintervention (repair or replacement) and development of greater than or equal to moderate LAVV dysfunction (defined as mean LAVV inflow gradient ≥8 mmHg and/or greater than or equal to moderate qualitative grade of LAVVR). Secondary outcomes included LAVV reintervention and greater than or equal to moderate LAVV dysfunction (either LAVVR or LAVVS).

### Definitions

Parachute LAVV was defined as a single left ventricular papillary muscle and no mural leaflet; *forme fruste* parachute LAVV was defined as 2 closely spaced papillary muscles and a diminutive mural leaflet. Transitional AVSD was defined by a small, restrictive ventricular septal defect, a primum atrial septal defect (ASD) and a common atrioventricular (AV) valve. Associated cardiac lesions involved assessment for additional ASDs or ventricular septal defects, coarctation of the aorta, double outlet right ventricle, left ventricular outflow tract obstruction, subaortic stenosis, aortic stenosis, persistent left superior vena cava to coronary sinus, sub-pulmonary/pulmonary stenosis, transposition of the great arteries and tetralogy of Fallot.

### Data collection

Patient medical records were used to extract demographics, anatomy, surgical characteristics and echocardiographic data. Echocardiographic data (including degree of LAVVS and LAVVR) were taken from preoperative transthoracic echocardiograms (TTE) and immediate postoperative transoesophageal echocardiograms. LAVVS was measured using mean LAVV inflow gradient on continuous-wave Doppler (mmHg) in the apical 4-chamber view with sample volume placed at the LAVV leaflet tips, and measurements were averaged over 3 beats. Left ventricle (LV) systolic dysfunction was defined as ejection fraction <55%. Exploratory TTE variables were assessed by a cardiologist blinded to outcomes and were used to quantify level of unbalance and leaflet pathology. All exploratory TTE variables were indexed to body surface area (BSA) either through calculating z-scores [17, 18] or (variable) BSA ratios. These variables include LV end-diastolic volume (LVEDV) z-score, atrioventricular valve index [19, 20], RV/LV inflow angle [19, 20], left AV area (subcostal short axis)/BSA, LAVV diameter/BSA, LAVV inflow diameter/BSA, left AV inflow diameter/total diameter [20] and right AV area (subcostal short axis)/BSA.

LAVV repair strategies were not uniform and were at the discretion of the operating surgeon. Primary repair strategies involved either single-patch, double-patch or modified single-patch for complete AVSDs, and primum ASD closure with or without ventricular septal defect closure for transitional canals. LAVV interventions included no, partial or complete cleft closure, papillary muscle splitting or mobilization, chordal division or placement, annuloplasty, leaflet patch augmentation and commissuroplasty. Concurrent repairs at the time of primary AV canal surgery were also recorded and included coarctation repair, fenestrated ASD closure, left ventricular outflow tract surgery and single-ventricle takedown. The need for multiple cardiopulmonary bypass runs was also recorded.

### Statistical analysis

Comparisons between presence and absence of an outcome were assessed using Student’s *t*-test or Wilcoxon rank sum test. For comparisons of categorical variables, a Fisher’s exact test was performed. Competing risks cumulative incidence functions were estimated to describe time-to-event for LAVV reintervention, death and greater than or equal to moderate LAVV dysfunction. Kaplan–Meier methodology was used to estimate freedom from the composite outcome. Predictor variables included those shown in Tables 1 and 2. Univariate associations between predictors and the component outcomes were assessed by subdistribution Cox proportional hazards regression in the competing risks framework [21] and Cox regression (for the composite outcome). For multivariable model construction for the composite outcome, stepwise selection was used, with a criterion for entry into the model of *P* < 0.15 and the criterion for remaining in the model was *P* < 0.05. A *P*-value < 0.05 was considered statistically significant. Analyses were performed using SAS 9.4 (SAS Institute, Inc., Cary, NC) and R version 4.1.2.

**Table 1:** Patient demographics and anatomy with univariate model results for time to the composite outcome.

		Composite outcome			
Variable	Overall (*n* = 36)	Yes (*n* = 16)	No (*n* = 20)	HR (95% CI)	*P*-value
Age at surgery (years)	0.34 (0.19–0.46)	0.31 (0.18–0.40)	0.41 (0.20–1.02)	0.25 (0.04–1.59)	0.14
Male	7 (19.4%)	3 (18.8%)	4 (20.0%)	1.55 (0.43, 5.55)	0.50
Weight at surgery (kg)	4.60 (3.80–5.70)	3.80 (3.36–5.22)	4.65 (4.50–7.20)	0.71 (0.48–1.05)	0.09
Known genetic disorder				0.45 (0.16–1.25)^a^	0.13
None	18 (50.0%)	10 (62.5%)	8 (40.0%)		
Trisomy 21	15 (41.7%)	5 (31.3%)	10 (50.0%)		
Heterotaxy	1 (2.8%)	1 (6.3%)	0 (0%)		
Other	2 (5.6%)	0 (0%)	2 (10.0%)		
AV canal type					0.13
Complete	27 (75.0%)	11 (68.8%)	16 (80.0%)	0.45 (0.16–1.25)	
Transitional	9 (25.0%)	5 (31.3%)	4 (20.0%)	Ref	
Parachute type					0.76
Complete parachute	21 (58.3%)	9 (56.3%)	12 (60.0%)	0.86 (0.32–2.31)	
*Forme fruste*	15 (41.7%)	7 (43.8%)	8 (40.0%)	Ref	
AV canal balance					0.26
Balanced	11 (30.6%)	3 (18.8%)	8 (40.0%)	0.49 (0.14–1.71)	
Right dominant	25 (69.4%)	13 (81.3%)	12 (60.0%)	Ref	
Associated lesions	35 (97.2%)	16 (100%)	19 (95.0%)	/	NA
Secundum ASD	18 (50.0%)	9 (56.3%)	9 (45.0%)	1.18 (0.44–3.19)	0.74
Additional VSD	2 (5.6%)	2 (12.5%)	0 (0%)	–	NA
Coarctation of the aorta	2 (5.6%)	2 (12.5%)	0 (0%)	–	NA
LVOT obstruction/concurrent subaortic stenosis/aortic stenosis	4 (11.1%)	2 (12.5%)	2 (10.0%)	0.87 (0.20–3.84)	0.85
Left superior vena cava to coronary sinus	6 (16.7%)	3 (18.8%)	3 (15.0%)	1.01 (0.29–3.56)	0.99

Data are given as median (interquartile range) or number (percentage).

a HR is for presence of a genetic disorder vs no presence of a genetic disorder.

ASD: atrial septal defect; AV: atrioventricular; CI: confidence interval; HR: hazard ratio; LVOT: left ventricular outflow tract; NA: not applicable; Ref: reference; VSD: ventricular septal defect.

**Table 2:** Clinical preoperative TTE variables, prior surgeries and AVSD repair strategy with univariate model results for time to the composite outcome.

		Composite outcome			
Variable	Overall (*n* = 36)	Yes (*n* = 16)	No (*n* = 20)	HR (95% CI)	*P*-value
Left ventricular ejection fraction (%)	56 (55–61)	56.1 (50–60)	56 (55–62)	0.95 (0.88–1.02)	0.17
Qualitative LV function				2.26 (0.78–6.55)^a^	0.13
Normal	30 (83.3%)	11 (68.8%)	19 (95.0%)		
Mild	5 (13.9%)	4 (25.0%)	1 (5.0%)		
Mild–moderate	0	0	0		
Moderate	0	0	0		
Moderate–severe	0	0	0		
Severe	1 (2.8%)	1 (6.3%)	0 (0%)		
AVVR degree					
None or trivial	3 (8.3%)	2 (12.5%)	1 (5.0%)		
Mild	17 (47.2%)	5 (31.3%)	12 (60.0%)		
Mild–moderate	10 (27.8%)	5 (31.3%)	5 (25.0%)		
Moderate	1 (2.8%)	1 (6.3%)	0 (0%)	1.89 (0.70–5.11)^b^	0.21
Moderate–severe	3 (8.3%)	1 (6.3%)	2 (10.0%)		
Surgical history				1.64 (0.59–4.54)	0.34
Coarctation repair	4 (11.1%)	2 (12.5%)	2 (10.0%)	1.15 (0.26–5.09)	0.85
Pulmonary artery band	7 (19.4%)	4 (25.0%)	3 (15.0%)	1.56 (0.50–4.86)	0.44
Patent ductus arteriosus ligation	3 (8.3%)	1 (6.3%)	2 (10.0%)	0.61 (0.08–4.61)	0.63
Stage I/bidirectional Glenn/hybrid	3 (8.3%)	2 (12.5%)	1 (5.0%)	2.78 (0.63–12.35)	0.18
Other	5 (13.9%)	2 (12.5%)	3 (15.0%)	0.83 (0.19–3.65)	0.80
Pre-op weight (kg)	4.45 (3.70, 6.00)	3.80 (3.47, 5.16)	4.60 (4.20, 7.16)	0.74 (0.52–1.05)	0.09
Pre-op height (cm)	56.5 (52.8, 62.0)	54.5 (52.0, 58.5)	57.8 (53.4, 72.3)	0.95 (0.90–1.01)	0.11
Pre-op BSA (m^2^)	0.27 (0.24, 0.33)	0.24 (0.23, 0.29)	0.28 (0.25, 0.38)	0.23 (0.00–34.85)	0.57
Repair strategy					
Single patch	3 (8.3%)	1 (6.3%)	2 (10.0%)	0.49 (0.06–3.74)	0.49
Double patch	23 (63.9%)	10 (62.5%)	13 (65.0%)	0.99 (0.36–2.74)	0.99
Modified single patch	1 (2.8%)	0 (0%)	1 (5.0%)	–	NA
Primum ASD closure	9 (25.0%)	5 (31.3%)	4 (20.0%)	1.69 (0.59–4.88)	0.33
VSD closure	8 (22.2%)	4 (25.0%)	4 (20.0%)	1.26 (0.41–3.93)	0.69
Other	0	0	0	–	NA
Degree of cleft closure					**0.02** ^c^
Full	9 (25.7%)	5 (31.3%)	4 (21.1%)	Ref	
Partial	23 (65.7%)	8 (50.0%)	15 (78.9%)	0.54 (0.18–1.65)	
None	3 (8.6%)	3 (18.8%)	0 (0%)	4.09 (0.87–19.25)	
LAVV repair techniques	27 (75.0%)	11 (68.8%)	16 (80.0%)	0.76 (0.27–2.20)	0.62
Papillary muscle splitting/moving	14 (38.9%)	5 (31.3%)	9 (45.0%)	0.64 (0.22–1.84)	0.41
Chord division	5 (13.9%)	1 (6.3%)	4 (20.0%)	0.30 (0.04–2.27)	0.24
Annuloplasty	3 (8.3%)	1 (6.3%)	2 (10.0%)	1.15 (0.15–8.74)	0.90
Leaflet patch augmentation	3 (8.3%)	2 (12.5%)	1 (5.0%)	1.48 (0.33–6.55)	0.61
Chord placement	1 (2.8%)	1 (6.3%)	0 (0%)	–	NA
Commissuroplasty	0	0	0	–	NA
Concurrent repairs	35 (97.2%)	16 (100%)	19 (95.0%)	–	NA
PDA ligation	12 (33.3%)	5 (31.3%)	7 (35.0%)	0.89 (0.31–2.56)	0.83
Coarctation repair	2 (5.6%)	2 (12.5%)	0 (0%)	–	NA
Tricuspid valve repair	28 (77.8%)	13 (81.3%)	15 (75.0%)	1.93 (0.55–6.83)	0.31
Fenestrated ASD closure	12 (33.3%)	7 (43.8%)	5 (25.0%)	1.40 (0.52–3.76)	0.51
LVOT intervention	4 (11.1%)	1 (6.3%)	3 (15.0%)	0.40 (0.05–3.07)	0.38
Damus–Kaye–Stansel/stage I/Glenn/Hybrid takedown	2 (5.6%)	1 (6.3%)	1 (5.0%)	1.39 (0.18–10.55)	0.75
PA band takedown	7 (19.4%)	4 (25.0%)	3 (15.0%)	1.56 (0.50–4.86)	0.44
Secundum ASD closure	15 (41.7%)	7 (43.8%)	8 (40.0%)	0.96 (0.36–2.58)	0.94
Other	17 (47.2%)	8 (50.0%)	9 (45.0%)	0.765	0.48
Multiple bypass runs during primary repair					0.76
Yes	4 (11.1%)	2 (12.5%)	2 (10.0%)	1.26 (0.28–5.54)	
No	32 (88.9%)	14 (87.5%)	18 (90.0%)	Ref	

a For normal versus abnormal.

b For greater than or equal to moderate versus less than moderate.

c Bold values are considered statistically significant (P-value < 0.05).

Frequency (%) or median (IQR) are displayed.

ASD: atrial septal defect; AVSD: atrioventricular septal defect; AVVR: AV valve regurgitation; CI: confidence interval; HR: hazard ratio; IQR: interquartile range; LAVV: left atrioventricular valve; LV: left ventricle; LVOT: left ventricular outflow tract; PA: pulmonary artery; PDA: patent ductus arteriosus; Ref: reference; TTE: transthoracic echocardiogram; VSD: ventricular septal defect.

## RESULTS

### Demographics

From 2012 to 2021, 36 AVSD patients with parachute LAVV underwent repair with baseline characteristics in Table 1. Overall, 21 (58%) had parachute LAVV and 15 (42%) had *forme fruste* parachute. Most patients had a complete AVSD (*n* = 27, 75%), while 9 (25%) had a transitional AVSD. The majority (*n* = 25, 69%) had right-dominant AVSD (7 with transitional AVSD and 18 with complete AVSD), while 11 had balanced AVSD (2 with transitional AVSD and 9 with complete AVSD). The median LVEDV z-score was 0.25 (interquartile range –1.63 to 0.58) and among those with right-dominant AV canals, 6 (24%) had a LVEDV z-score < –2.

Median age at primary AVSD repair was 4.1 months (interquartile range 2.3–5.5 months). Most patients (75%) had mild or mild-to-moderate AV valve regurgitation (AVVR) before primary repair. Surgical repair strategies are outlined in Table 2. In patients with complete AVSD, 23 had two-patch repair, 3 had single-patch and 1 modified single-patch (Australian) repair. The cleft was partially closed in 23 patients (64%), completely closed in 9 patients (25%) and left open in 3 patients (8%). Fourteen patients had splitting of a papillary muscle (39%) and 5 (14%) underwent division of LAVV chordae.

### Outcomes

**Figure 1:**
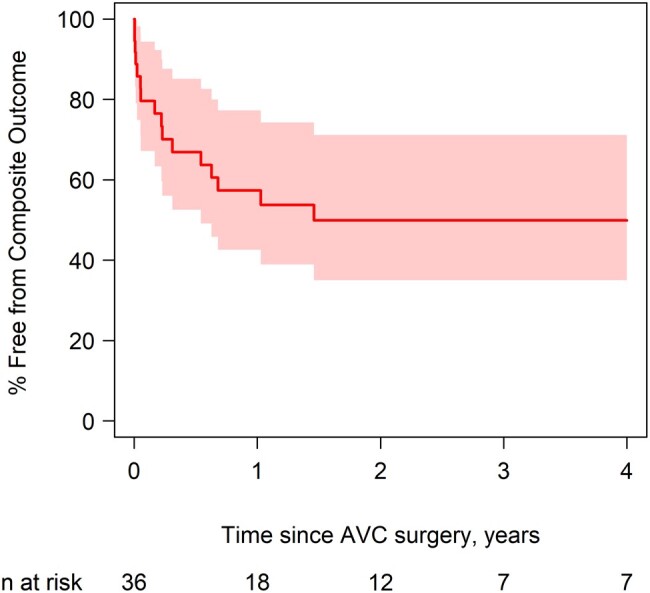
Estimated Kaplan–Meier freedom from composite outcome with pointwise 95% confidence bands. No events after 2 years. Kaplan-Meier Freedom from Composite Outcome.

Among those who survived, median follow-up was 2.6 years (interquartile range 1.0–5.6 years). Sixteen (44%) patients met the composite outcome with 3 deaths, 6 LAVV reinterventions and 14 patients who developed greater than or equal to moderate LAVVR or a LAVVS gradient ≥8 mmHg. Freedom from the composite outcome (Fig. 1) declined rapidly over the 1st year following AVSD repair with 67% at 6 months and 50% at 1.5 years. All patients who experienced the composite outcome did so within 2 years of primary AVSD repair. Degree of cleft closure was the only statistically significant predictor of the composite in multivariate analysis. No cleft closure was associated with increased risk of experiencing the composite outcome when compared to partial or complete cleft closure in both univariate [hazard ratio (HR) 4.09, *P* = 0.02] and multivariate analysis (HR 7.62, *P* = 0.02) (Table 2, Supplementary Material, Table S1).

**Table:** 

Time since surgery	% Free from Composite Outcome (95% CI)
0.5 years	67 (48–80)
1 year	57 (39–72)
1.5 years	50 (31–66)
5 years	50 (31–66)


Among the 3 patients who had no cleft closure, 2 had complete AVSD (1 balanced, 1 right-dominant) and 1 had transitional, right-dominant AVSD. Two patients had mild–moderate AVVR prior to surgery and 1 had severe AVVR requiring hospitalization with inotropes prior to surgery. One patient underwent papillary muscle splitting during primary repair, and another required posterior annuloplasty to better approximate the mural leaflet to the inferior bridging leaflet.

All 3 repairs only required 1 bypass run, and there was no initial attempt at cleft closure (as it was felt that the valve orifice in all 3 patients precluded closure). One patient died, 1 developed LAVVR alone and 1 developed LAVVR requiring 2 sequential LAVV repairs on postop days 5 and 6 to correct it. The repairs 1st involved suture cleft closure and annuloplasty followed by patch cleft closure and placement of artificial chordae.

**Figure 2:**
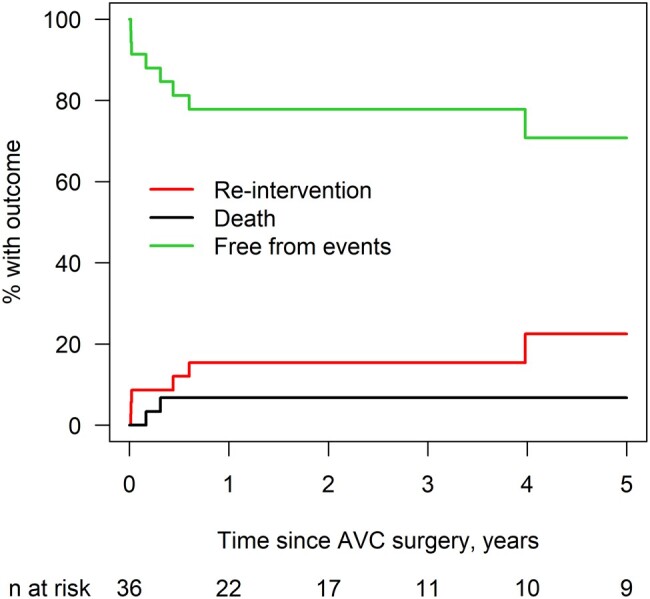
Cumulative incidence of LAVV reintervention (6 events) and death (2 events). No events occurred after 5 years. LAVV: left atrioventricular valve. Cumulative incidence.

Figure 2 displays the competing risk cumulative incidence of death and LAVV reintervention over 5 years post AVSD repair. Six patients (18%) underwent LAVV reintervention (5 surgical and 1 catheter balloon dilation), resulting in LAVV reintervention incidence of 15% at 1 and 3 years. All 6 patients had greater than or equal to moderate LAVV dysfunction (3 with greater than or equal to moderate LAVVR, 2 with greater than or equal to moderate LAVVS and 1 with both greater than or equal to moderate LAVVR and LAVVS) prior to reintervention. All 6 patients who underwent LAVV reintervention had right-dominant AVSD.

**Table:** 

Time since surgery	% Reintervention (95% CI)	% death (95% CI)
3 months	8.7 (2.2–21.0)	3.4 (0.2–15.0)
6 months	12.0 (3.7–25.7)	6.8 (1.1–19.7)
9 months	15.4 (5.5–30.1)	6.8 (1.1–19.7)
1 year	15.4 (5.5–30.1)	6.8 (1.1–19.7)
3 years	15.4 (5.5–30.1)	6.8 (1.1–19.7)
5 years	22.5 (7.7–42.0)	6.8 (1.1–19.7)


Of the 6 LAVV reinterventions, 3 occurred within 2 weeks of primary AVSD repair, while 5 occurred within 8 months. Of these patients, 4 underwent more than 1 reintervention and 3 ultimately had a LAVV replacement. Two of these patients underwent an initial surgical LAVV repair followed by replacement. One patient underwent 3 LAVV reinterventions prior to replacement. Significant comorbidities were associated with replacement, with 1 death, 2 pacemaker placements and 1 patient with subsequent Melody valve balloon dilation and need for additional valve replacement surgery.

During follow-up, there were 3 deaths, 2 of which occurred within 3 months of primary AVSD repair and without postoperative LAVV dysfunction or reintervention. One death occurred after LAVV reintervention and subsequent replacement.

Factors associated with LAVV reintervention in univariate analysis were transitional AVSD (vs complete AVSD) (HR 7.69, *P* = 0.01), a history of initial single-ventricle palliation prior to full AVSD repair (HR 11.57, *P* = 0.007) and greater than or equal to moderate AVVR on preoperative TTE (HR 8.09, *P* = 0.02) (Tables 3 and 4). All 6 patients who underwent LAVV reintervention had right-dominant AVSD. LAVV function on immediate postoperative transoesophageal echocardiogram was analysed by qualitative degree of LAVVR and LAVVS mean gradient. These values were not predictive of LAVV reintervention.

**Table 3:** Patient demographics and anatomy with univariate model results for time to LAVV reintervention.

		Reintervention			
Variable	Overall (*n* = 36)	Yes (*n* = 6)	No (*n* = 30)	HR (95% CI)	*P*-value
Age at surgery (years)	0.34 (0.19–0.46)	0.28 (0.10–0.44)	0.34 (0.19–0.50)	0.16 (0.01–4.40)	0.28
Male	7 (19.4%)	0 (0%)	7 (23.3%)	/	NA
Weight at surgery (kg)	4.6 (3.8–5.7)	4.3 (3.4–5.4)	4.6 (3.8–6.8)	0.78 (0.48–1.26)	0.31
Known genetic disorder				0.16 (0.02–1.49)^a^	0.11
None	18 (50.0%)	5 (83.3%)	13 (43.3%)		
Trisomy 21	15 (41.7%)	1 (16.7%)	14 (46.7%)		
Heterotaxy	1 (2.8%)	0 (0%)	1 (3.3%)		
AV canal type					**0.01** ^b^
Complete parachute	27 (75.0%)	2 (33.3%)	25 (83.3%)	Ref	
Transitional	9 (25.0%)	4 (66.7%)	5 (16.7%)	7.69 (1.56–33.33)	
Parachute type					0.30
Complete	21 (58.3%)	5 (83.3%)	16 (53.3%)	3.15 (0.36–27.37)	
Forme fruste	15 (41.7%)	1 (16.7%)	14 (46.7%)	Ref	
AV canal balance					NA
Balanced	11 (30.6%)	0 (0%)	11 (36.7%)	/	
Right dominant	25 (69.4%)	6 (100%)	19 (63.3%)	/	
Associated lesions	35 (97.2%)	6 (100%)	29 (96.7%)	/	NA
ASD	18 (50.0%)	4 (66.7%)	14 (46.7%)	2.01 (0.41–9.95)	0.39
VSD	2 (5.6%)	0 (0%)	2 (6.7%)	–	NA
Coarctation of the aorta	2 (5.6%)	2 (33.3%)	0 (0%)	–	NA
LVOT obstruction/concurrent subaortic stenosis/aortic stenosis	4 (11.1%)	0 (0%)	4 (13.3%)	–	NA
Left superior vena cava to coronary sinus	6 (16.7%)	0 (0%)	6 (20.0%)	–	NA

Frequency (%) or median (IQR) are displayed.

a HR is for presence of a genetic disorder versus no presence of a genetic disorder.

b Bold values are considered statistically significant (P-value < 0.05)

ASD: atrial septal defect; CI: confidence interval; HR: hazard ratio; IQR: interquartile range; LAVV: left atrioventricular valve; LVOT: left ventricular outflow tract; NA: not applicable; Ref: reference; VSD: ventricular septal defect.

**Table 4:** Clinical preoperative TTE variables, prior surgeries, and AVSD repair strategy with univariate model results for time to LAVV reintervention.

		Reintervention			
Variable	Overall (*n* = 36)	Yes (*n* = 6)	No (*n* = 30)	HR (95% CI)	*P* value
Left ventricular ejection fraction (%)	56 (55–61)	58.50 (46–65)	56 (55–59.10)	0.96 (0.84–1.09)	0.51
Qualitative LV function				2.24 (0.40–12.46)^a^	0.36
Normal	30 (83.3%)	4 (66.7%)	26 (86.7%)	Ref	
Mild	5 (13.9%)	1 (16.7%)	4 (13.3%)		
Mild–moderate	0	0	0		
Moderate	0	0	0		
Moderate–severe	0	0	0		
Severe	1 (2.8%)	1 (16.7%)	0 (0%)		
AVVR degree					
None or trivial	3 (8.3%)	0 (0%)	3 (10.0%)		
Mild	17 (47.2%)	1 (16.7%)	16 (53.3%)		
Mild–moderate	10 (27.8%)	3 (50.0%)	7 (23.3%)		
Moderate	1 (2.8%)	0 (0%)	1 (3.3%)	8.09 (1.35–48.56)^b^	**0.02** ^d^
Moderate–severe	3 (8.3%)	1 (16.7%)	2 (6.7%)		
Surgical history	11 (30.6%)	3 (50.0%)	8 (26.7%)	2.30 (0.49–10.78)	0.29
Coarctation repair	4 (11.1%)	1 (16.7%)	3 (10.0%)	1.25 (0.22–7.28)	0.80
Pulmonary artery band	7 (19.4%)	1 (16.7%)	6 (20.0%)	0.73 (0.11–5.12)	0.76
Patent ductus arteriosus ligation	3 (8.3%)	0 (0%)	3 (10.0%)	/	NA
Stage I/bidirectional Glenn/Hybrid	3 (8.3%)	2 (33.3%)	1 (3.3%)	11.57 (1.92–69.62)	**0.007**
Other	5 (13.9%)	1 (16.7%)	4 (13.3%)	1.28 (0.13–12.35)	0.83
Pre-op weight (kg)	4.45 (3.70, 6.00)	3.75 (3.34, 5.40)	4.55 (3.80, 6.80)	0.74 (0.43–1.27)	0.27
Pre-op height (cm)	56.5 (52.8, 62.0)	53.5 (52.0, 58.5)	57.3 (53.0, 64.0)	0.94 (0.87–1.01)	0.12
Pre-op BSA (m^2^)	0.27 (0.24, 0.33)	0.24 (0.22, 0.33)	0.27 (0.24, 0.32)	1.20 (0.05–27.65)^c^	0.91
Repair strategy					
Single patch	3 (8.3%)	0 (0%)	3 (10.0%)	–	NA
Double patch	23 (63.9%)	2 (33.3%)	21 (70.0%)	0.25 (0.05–1.21)	0.08
Modified single patch	1 (2.8%)	0 (0%)	1 (3.3%)	–	NA
Primum ASD closure	9 (25.0%)	4 (66.7%)	5 (16.7%)	7.68 (1.55–37.98)	**0.01**
VSD closure	8 (22.2%)	3 (50.0%)	5 (16.7%)	4.16 (0.89–19.53)	0.07
Degree of cleft closure					0.67
Full	9 (25.7%)	2 (33.3%)	7 (24.1%)	0.75 (0.06–9.48)	
Partial	23 (65.7%)	3 (50.0%)	20 (69.0%)	0.39 (0.03–4.41)	
None	3 (8.6%)	1 (16.7%)	2 (6.9%)	Ref	
LAVV repair techniques	27 (75.0%)	5 (83.3%)	22 (73.3%)	1.95 (0.24–16.06)	0.54
Papillary muscle splitting/moving	14 (38.9%)	1 (16.7%)	13 (43.3%)	0.29 (0.03–2.50)	0.26
Chord division	5 (13.9%)	1 (16.7%)	4 (13.3%)	0.92 (0.15–5.43)	0.92
Annuloplasty	3 (8.3%)	1 (16.7%)	2 (6.7%)	2.87 (0.30–27.30)	0.36
Leaflet patch augmentation	3 (8.3%)	1 (16.7%)	2 (6.7%)	2.19 (0.26–18.51)	0.47
Chord placement	1 (2.8%)	1 (16.7%)	0 (0%)	–	NA
Concurrent repairs	35 (97.2%)	6 (100%)	29 (96.7%)	–	NA
Coarctation repair	2 (5.6%)	2 (33.3%)	0 (0%)	–	NA
Tricuspid valve repair	28 (77.8%)	5 (83.3%)	23 (76.7%)	2.14 (0.25–18.09)	0.48
Fenestrated ASD closure	12 (33.3%)	2 (33.3%)	10 (33.3%)	0.94 (0.17–5.16)	0.94
LVOT intervention	4 (11.1%)	0 (0%)	4 (13.3%)	–	NA
Damus–Kaye–Stansel/stage 1/Glenn/Hybrid takedown	2 (5.6%)	1 (16.7%)	1 (3.3%)	4.71 (0.62–35.70)	0.13
PA band takedown	7 (19.4%)	1 (16.7%)	6 (20.0%)	0.73 (0.11–5.12)	0.76
Secundum ASD closure	15 (41.7%)	3 (50.0%)	12 (40.0%)	1.24 (0.27–5.80)	0.78
Other	17 (47.2%)	3 (50.0%)	14 (46.7%)	1.36 (0.29–6.51)	0.70
Multiple bypass runs					0.72
Yes	4 (11.1%)	1 (16.7%)	3 (10.0%)	1.54 (0.15–16.27)	
No	32 (88.9%)	5 (83.3%)	27 (90.0%)	Ref	

Frequency (%) or median (IQR) are displayed.

a For normal versus abnormal.

b For greater than or equal to moderate versus less than moderate.

c Per 0.1 unit increase.

d Bold values are considered statistically significant (P-value < 0.05)

ASD: atrial septal defect; AVSD: atrioventricular septal defect; AVVR: AV valve regurgitation; CI: confidence interval; HR: hazard ratio; IQR: interquartile range; LAVV: left atrioventricular valve; LV: left ventricle; LVOT: left ventricular outflow tract; NA: not applicable; PA: pulmonary artery; Ref: reference; TTE: transthoracic echocardiogram; VSD: ventricular septal defect.

**Figure 3:**
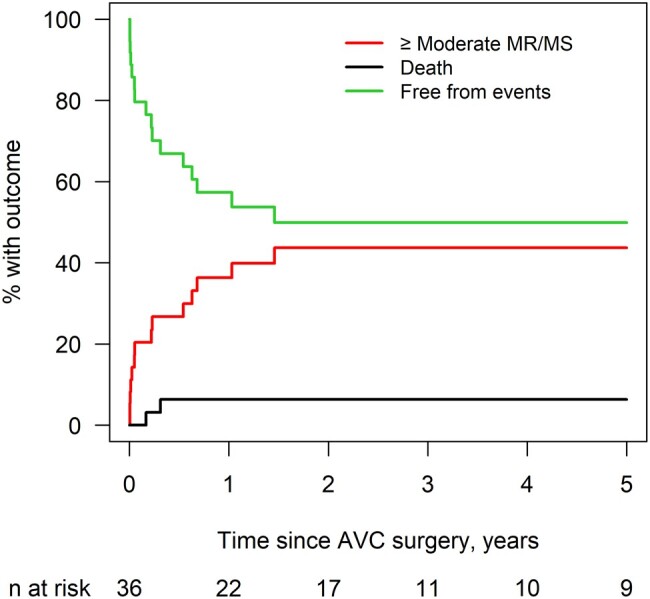
Cumulative Incidence of at least moderate LAVVR/LAVVS (14 events), and death (2 events). No events occurred after 5 years. LAVVR: left atrioventricular valve regurgitation; LAVVS: left atrioventricular valve stenosis. Cumulative incidence.

Competing risk curves in Fig. 3 show that by 1 year, the risk for development of greater than or equal to moderate LAVV dysfunction (LAVVS and/or LAVVR) is 36% and 44% by 3 years. Overall, 14 (39%) patients developed greater than or equal to moderate LAVV dysfunction (5 with greater than or equal to moderate LAVVR, 5 with greater than or equal to moderate LAVVS and 4 with both greater than or equal to moderate LAVVS and LAVVR). Univariate risk factors for greater than or equal to moderate LAVVS and/or LAVVR were younger age at surgery (HR 0.20; CI 0.05–0.82; *P* = 0.025), lower weight at surgery (HR 0.67; CI 0.47–0.96, *P* = 0.030) and preoperative LV dysfunction (HR 2.97; CI 1.22–7.27; *P* = 0.017).

**Table:** 

Time since surgery	% Moderate MR/MS (95% CI)	% death (95% CI)
3 months	26.8 (13.1–42.6)	3.2 (0.2–14.3)
6 months	26.8 (13.1–42.6)	6.4 (1.1–18.7)
9 months	36.3 (20.1–52.7)	6.4 (1.1–18.7)
1 year	36.3 (20.1–52.7)	6.4 (1.1–18.7)
3 years	43.7 (25.7–60.4)	6.4 (1.1–18.7)
5 years	43.7 (25.7–60.4)	6.4 (1.1–18.7)


Of the exploratory TTE variables collected, none were predictive of the outcomes (Supplementary Material, Tables S2 and S3).

## DISCUSSION

In this study, we describe contemporary, medium-term outcomes after full repair of AVSD with parachute LAVV. This remains a high-risk population with 44% of patients having greater than or equal to moderate valve dysfunction, LAVV reintervention or death within 18 months of surgery. We also found that the majority of LAVV reinterventions were performed within 8 months of primary repair, and all LAVV reinterventions were performed in patients with a right-dominant AVSD. Leaving the cleft open was independently associated with increased risk of the composite of death, LAVV dysfunction and reintervention.

Despite continued evolution and improvement in surgical techniques, we found a high burden of LAVV reintervention and dysfunction over follow-up. Myers *et al.* from Boston Children’s Hospital previously analysed our institutional experience with biventricular repair in patients with complete AVSDs and parachute LAVV from 2001 to 2012. Our cohort had a similar freedom from LAVV reintervention 1 year post AVSD repair but a higher freedom from LAVV reintervention at 3 years. Both of our cohorts were skewed towards high-risk patients as evidenced by the high prevalence of unbalanced, right-dominant AVSD patients (69% of included patients in this study vs 67% in Myers study). Overall, this suggests a modest incremental improvement in reoperation rates between surgical eras. Furthermore, all LAVV reinterventions in the present study occurred in patients with unbalanced, right-dominant canal defects, which is concordant with Myers *et al.* who showed that an indexed LVEDV <20 ml/m^2^ resulted in statistically significant lower survival and increased proportion of LAVV reintervention.

The alternative management strategy for right-dominant AVSD patients is single-ventricle palliation. Although the LAVV reintervention rate in our cohort is relatively high, medium-term survival was >90%. These mortality and reintervention rates compare favourably to single-ventricle palliation outcomes, with prior publications showing high mortality (40%) and reintervention (60%) in unbalanced AVSD patients undergoing single-ventricle palliation [15]. These outcome data support the concept of primary biventricular repair or biventricular conversion as compared with single-ventricle palliation in this patient group when the LV is adequate in size but also emphasizes that there is significant risk of LAVV reintervention [10, 15, 22–24, 25].

Management of the cleft in AVSD patients, particularly in those with parachute-type LAVV, remains a challenge. Complete, and, in some cases, partial cleft closure is not feasible in patients with parachute LAVV as this would result in minimal inflow orifice and significant stenosis, while leaving the cleft completely open has been identified as a risk factor for development of postoperative LAVVR in prior studies [8–10]. Over the last 30–40 years, there has been a gradual evolution towards closing the cleft whenever possible, even in the setting of an abnormal LAVV. Data from the Mayo Clinic analysed long-term outcomes of surgical repair of 28 AVSD patients with parachute LAVV from 1977 to 2010 [7]. In this earlier era, the cleft was typically left open, and LAVVR was common and frequently led to reoperation. In the cohort from 2001 to 2012 at our institution in which the cleft was generally closed as much as deemed possible, Myers *et al.* described results like ours with indications for LAVV reintervention in their cohort distributed among LAVVR (60%), LAVVS (20%) or mixed valve disease (20%). The increase in proportion of patients undergoing LAVV reintervention for LAVVS or mixed LAVV disease in both our study and Myers *et al.* is likely related to the trend towards more aggressive cleft closure in the contemporary management of this patient population.

In our study, leaving the cleft completely open was independently associated with the composite outcome of death, reintervention and LAVVS and/or LAVVR. In this small cohort, it is difficult to determine if this finding is related to severity and complexity of valvar disease and LAVV hypoplasia versus surgical technique.

Therefore, larger, multicentre studies with central review of imaging data are needed to further explore how surgical management of the cleft influences medium and long-term outcomes in this patient population.

When managing a cleft deemed ineligible for suture closure, our institutional practice over the study duration was to close the cleft as fully as possible based on surgeon’s judgement and to utilize several strategies at both the base of the cleft and the papillary muscle. Decreasing annular dimension at the base of the cleft can reduce tension and decrease the chance of regurgitation. Furthermore, gently inverting or folding the superior and inferior bridging leaflets down into the ventricle can create more coaptation height at the cleft base. The papillary muscle is generally split to improve LAVV inflow, but this can come at the cost of splaying of the leaflets along the cleft and subsequent LAVVR. There is no specific rule for the degree of PM splitting, but one must balance opening up the inflow without predisposing to LAVVR.

Existing data on echocardiographic predictors of clinical outcomes for AVSD patients with parachute LAVV are scant. We found that greater than or equal to moderate AVVR prior to repair was associated with increased risk of LAVV reintervention. We evaluated a number exploratory preoperative TTE variables but found no significant associations with LAVV dysfunction or reintervention.

### Limitations

This study is limited by its single-centre, retrospective design. Because of the small sample and small number of primary outcome events, as well as patient heterogeneity, our study is underpowered to detect some associations that may be clinically significant. For this reason, future studies with analysis of echocardiographic predictors in a larger cohort of patients may be of interest. Additionally, follow-up times are short, there is no universal indication for LAVV reintervention following AV canal repair, and there is a lack of consensus in surgical management of the LAVV (particularly management of the LAVV cleft).Finally, surgical techniques have evolved over the study period and differ by surgeon.

## CONCLUSIONS

Repair of AVSD with parachute LAVV remains a clinical challenge with a high rate of LAVV reintervention and valvar dysfunction in medium-term follow-up. All LAVV reinterventions in patients with right-dominant AVSDs, but low postoperative mortality when compared to single-ventricle palliation supports biventricular repair whenever possible. Leaving the LAVV cleft open during primary AVSD repair might be an independent predictor of poorer overall outcomes and should be avoided when permitted by valvar anatomy. Multicentre cohort studies and registries are needed to determine independent predictive variables associated with outcomes in this patient group.

## Supplementary Material

ivae048_Supplementary_Data

## Data Availability

The data underlying this article will be shared on reasonable request to the corresponding author.

## Notes

Presented at the American College of Cardiology Conference, New Orleans, LA, USA, 5 March 2023. Abstract has not been submitted for the EACTS or ESTS annual meeting.

## ABBREVIATIONS

ASD: Atrial septal defect
AVSD: Atrioventricular septal defect
BSA: Body surface area
HR: Hazard ratio
LAVV: Left atrioventricular valve
LAVVR: Left atrioventricular valve regurgitation
LAVVS: Left atrioventricular valve stenosis
LV: Left ventricle
LVEDV: Left ventricular end-diastolic volume
TTE: Transthoracic echocardiogram
