# Supplementary material for: Cleft closure and other predictors of contemporary outcomes after atrioventricular canal repair in patients with parachute left atrioventricular valve
Source: Interdiscip Cardiovasc Thorac Surg. 2024 Mar 27;38(4):ivae048. doi: 10.1093/icvts/ivae048 (PMC11014788; doi:10.1093/icvts/ivae048)
Supplement: ivae048_Supplementary_Data [file ivae048_supplementary_data.docx]

**Supplementary Table 1:** Multivariable associations with time to the composite outcome.

|  |  | **Composite outcome** | |  |  |  |
| --- | --- | --- | --- | --- | --- | --- |
| **Variable** | **Overall (n=36)** | **Yes (n=16)** | **No (n=20)** | **Univariate HR (95% CI)** | **Multivariate HR (95% CI)** | **P value** |
| Degree of cleft closure |  |  |  |  |  | **0.024** |
| Full | 9 (25.7%) | 5 (31.3%) | 4 (21.1%) | Ref | 1.86 (0.61, 5.71) |  |
| Partial | 23 (65.7%) | 8 (50.0%) | 15 (78.9%) | 0.54 (0.18, 1.65) | Ref |  |
| None | 3 (8.6%) | 3 (18.8%) | 0 (0%) | 4.09 (0.87, 19.25) | 7.62 (1.77, 32.83) |  |

| **Supplementary Table 2:** Exploratory preoperative TTE variables with univariate model results for time to the composite outcome. | | | | | |
| --- | --- | --- | --- | --- | --- |
|  |  |  | |  |  |
|  |  | **Composite outcome** | |  |  |
| **Variable** | **Overall (n=36)** | **Yes (n=16)** | **No (n=20)** | **Univariate HR (95% CI)** | **P value** |
| LVEDV z-score | -0.25 (-1.63, 0.58) | -0.14 (-1.65, 1.80) | -0.51 (-1.43, 0.46) | 1.10 (0.81, 1.49) | 0.56 |
| LVEDV score < -2 |  |  |  |  | 0.95 |
| Yes | 7 (20.0%) | 3 (20.0%) | 4 (20.0%) | 1.04 (0.29, 3.69) |  |
| No | 28 (80.0%) | 12 (80.0%) | 16 (80.0%) | Ref |  |
| AVVI | 0.35 (0.28, 0.38) | 0.32 (0.27, 0.41) | 0.35 (0.32, 0.38) | 0.87 (0.42, 1.79)* | 0.71 |
| RV/LV inflow angle | 95 (72, 103) | 85 (69, 95) | 99.50 (80, 106) | 0.77 (0.57, 1.03)** | 0.08 |
| Left AV area (subcostal short axis)/BSA | 5.21 (3.54, 6.10) | 5.09 (3.00, 6.09) | 5.22 (3.72, 6.13) | 1.02 (0.74, 1.41) | 0.90 |
| Left AV valve diameter/BSA | 3.95 (2.81, 4.69) | 3.95 (3.43, 4.28) | 3.71 (2.60, 4.70) | 1.02 (0.69, 1.51) | 0.93 |
| Left AV valve inflow diameter/BSA | 1.96 (1.40, 2.57) | 1.96 (1.45, 2.38) | 1.87 (1.40, 2.67) | 0.84 (0.44, 1.61) | 0.60 |
| Left AV valve inflow/total diameter | 0.35 (0.29, 0.41) | 0.33 (0.29, 0.41) | 0.37 (0.30, 0.43) | 0.17 (0.00, 260.08) | 0.64 |
| Right AV area (subcostal short axis)/BSA | 9.65 (7.14, 11.72) | 8.75 (7.21, 13.22) | 9.68 (7.01, 11.49) | 1.06 (0.89, 1.26) | 0.55 |

*Per 0.1 unit increase

**Per 10-unit increase

**Supplementary Table 3:** Exploratory preoperative TTE variables with univariate model results for time to re-intervention.

|  |  | **Re-intervention** | | |  |  |
| --- | --- | --- | --- | --- | --- | --- |
| **Variable** | **Overall (n=36)** | **Yes (n=6)** | **No (n = 30)** | | **HR (95% CI)** | **P value** |
| LVEDV z-score | -0.25 (-1.63, 0.58) | -0.50 (-3.10, 0.48) | | -0.20 (-1.52, 0.58) | 0.85 (0.53, 1.36) | 0.51 |
| LVEDV z-score < -2 |  |  | |  |  | 0.28 |
| Yes | 7 (20.0%) | 2 (33.3%) | | 5 (17.2%) | 2.65 (0.45, 15.66) |  |
| No | 28 (80.0%) | 4 (66.7%) | | 24 (82.8%) | ref |  |
| AVVI | 0.35 (0.28, 0.38) | 0.34 (0.32, 0.41) | | 0.35 (0.28, 0.38) | 1.41 (0.39, 5.03) * | 0.60 |
| RV/LV inflow angle | 95 (72, 103) | 93 (69, 103) | | 95 (73, 103) | 0.99 (0.94, 1.03) | 0.58 |
| Left AV area (subcostal short axis)/BSA | 5.21 (3.54, 6.10) | 5.48 (3.00, 6.09) | | 5.21 (3.68, 6.10) | 1.02 (0.64, 1.60) | 0.95 |
| Left AV valve diameter/BSA | 3.95 (2.81, 4.69) | 3.91 (3.71, 4.18) | | 4.00 (2.81, 4.71) | 0.83 (0.57, 1.20) | 0.33 |
| Left AV valve inflow diameter/BSA | 1.96 (1.40, 2.57) | 1.37 (1.14, 1.96) | | 2.00 (1.42, 2.57) | 0.52 (0.19, 1.36) | 0.18 |
| Left AV valve inflow/total diameter | 0.35 (0.29, 0.41) | 0.33 (0.29, 0.35) | | 0.36 (0.30, 0.42) | 0.62 (0.28, 1.40)* | 0.25 |
| Right AV area (subcostal short axis)/BSA | 9.65 (7.14, 11.72) | 8.83 (6.37, 13.22) | | 9.65 (7.90, 11.26) | 0.99 (0.71, 1.38) | 0.96 |

*Per 0.1 unit increase
